# Deciphering the Complexity of the Immune Cell Landscape in Kidney Allograft Rejection

**DOI:** 10.3389/ti.2024.13835

**Published:** 2024-12-11

**Authors:** George Terinte-Balcan, Emilie Lebraud, Julien Zuber, Dany Anglicheau, Gener Ismail, Marion Rabant

**Affiliations:** ^1^ Nephrology department, “Carol Davila” University of Medicine and Pharmacy, Bucharest, Romania; ^2^ Department of Pathology, Necker-Enfants Malades Hospital, Assistance Publique—Hopitaux de Paris, Paris, France; ^3^ Centre National de la Recherche Scientifique (CNRS), Inserm U1151, Institut Necker-Enfants Malades, Université Paris Cité, Paris, France; ^4^ Department of Kidney and Metabolic Diseases, Transplantation and Clinical Immunology, Necker Hospital, Assistance Publique Hôpitaux de Paris (AP-HP), Paris, France; ^5^ Department of Nephrology, Fundeni Clinical Institute, Bucharest, Romania

**Keywords:** rejection, immune cells, infiltrates, heterogeneity, complexity

## Abstract

While the Banff classification dichotomizes kidney allograft rejection based on the localization of the cells in the different compartments of the cortical kidney tissue [schematically interstitium for T cell mediated rejection (TCMR) and glomerular and peritubular capillaries for antibody-mediated rejection (AMR)], there is a growing evidences that subtyping the immune cells can help refine prognosis prediction and treatment tailoring, based on a better understanding of the pathophysiology of kidney allograft rejection. In the last few years, multiplex IF techniques and automatic counting systems as well as transcriptomics studies (bulk, single-cell and spatial techniques) have provided invaluable clues to further decipher the complex puzzle of rejection. In this review, we aim to better describe the inflammatory infiltrates that occur during the course of kidney transplant rejection (active AMR, chronic active AMR and acute and chronic active TCMR). We also discuss minor components of the inflammatory response (mastocytes, eosinophils, neutrophils, follicular dendritic cells). We conclude by discussing whether the over simplistic dichotomy between AMR and TCMR, currently used in clinical routine, remains relevant given the great diversity of immune actors involved in rejections.

## Introduction

Transplantation remains the most efficient method of treating chronic kidney disease despite the high risk of adverse events such as rejection [1], infection or recurrence of disease. Regarding rejection, there is a growing consensus among the transplant community that the phenotypes are increasingly subtler and more complex [2]. The diagnosis of rejection is based on the Banff classification which takes into account (among other criteria) the lesions encountered in the graft such as glomerulitis, peritubular capillaritis, interstitial inflammation, tubulitis or arteritis [3, 4]. While the probability of being diagnosed with an episode of rejection remains relatively high at around 10%, considerable progress has been made in the last few years in reducing episodes of acute T-cell mediated rejections (TCMR) due to efficient therapy [5]. On the other hand, antibody-mediated rejection (AMR) still remains an unsolved problem with studies showing that chronic lesions (transplant glomerulopathy) are a major cause of late graft loss [6, 7]. One possible explanation for this mechanism could be that current therapies are predominantly focusing on T lymphocytes, while other cell types (for example, NK cells and macrophages) and soluble factors (complement) that participate to the immune response are currently being insufficiently targeted [8, 9].

During the course of rejection, there is a very complex interaction between different immunological mechanisms and immune cell types [10]. Interestingly, both innate and adaptative immune cells participate to the cascade of events leading to rejection [11]. In the first report addressing the heterogeneity of cell populations involved in mixed rejection (i.e., both TCMR and AMR), using single cell RNA-sequencing, Wu et al. found up to 16 different immune and renal stromal cell types [10]. Among the immune cell category, the group described 2 types of monocytes, T cells, B cells, plasma cells and mast cells [10]. More recently, Lamarthée et al. studied 16 biopsies using the same technique and also identified 10 different immune cell clusters including various subtypes of CD4^+^ T cells, CD8^+^ T cells, NK cells, dendritic cells, CD19^+^ B cells and monocytes/macrophages [12].

Although the Banff classification does not take into account the cellular composition of the inflammatory infiltrate, but only its intensity and localization (schematically within the interstitium for TCMR and within the capillaries for AMR), there is a growing belief that subtyping the leukocytes can help refine prognosis prediction and treatment tailoring, based on a better understanding of the pathophysiology of kidney allograft rejection [3, 13–17]. However, many hurdles have to be overcome in order to accurately identify and count cells in the setting of rejection. As a matter of fact, manually counting cells stained either by immunohistochemistry (IHC) or by immunofluorescence (IF) is very time consuming and raises the challenge of reproducibility [18, 19]. In the last few years, multiplex IF techniques and automatic counting systems have led to a large number of papers that focused on better describing the nature of the inflammatory infiltrates during rejection [20, 21]. Moreover, transcriptomics studies (bulk, single-cell and spatial techniques) have provided invaluable clues to further decipher the complex puzzle of rejection [10, 22, 23]. This is of particular importance in the setting of AMR where the pathophysiology becomes more complex with the description of antibody-independent and non-HLA donor specific antibody (DSA) mechanisms [9, 24].

In this review we aim to better describe the inflammatory infiltrate that occurs during the course of kidney transplantation, highlighting the different immune cell types involved and also their repartition. Therefore, we structured this review into active AMR, chronic active AMR and acute and chronic active TCMR. We also included a category of minor components of the inflammatory response that do not perfectly fit in the categories described. We conclude by discussing whether the over simplistic dichotomy between AMR and TCMR, currently used in clinical routine, remains relevant given the great diversity of immune actors involved in rejections.

## Active AMR

Regarding the diagnosis of AMR, glomerulitis represents an important component of microvascular inflammation (MVI) [3]. Glomerulitis is associated with the infiltration of different cell types, with the most common being macrophages and T lymphocytes [19, 25]. Interestingly, it has been shown using IHC that the mean number of monocytes per glomeruli is higher in C4d positive-AMR compared to C4d negative-AMR, whereas T cells are predominant in the glomeruli, in C4d negative AMR [15]. This finding was confirmed by another center that used electron microscopy [26]. Moreover, using IHC for CD68, Tinckam et al. demonstrated that a mean glomerular monocyte infiltration ≥1 was associated with a worse graft survival and independently predicted graft function at 2 and 4 years independent of C4d status [27]. More recently, Mölne et al. developed a Glomerular Macrophage Index (GMI) using IHC as the mean number of macrophages in 10 glomeruli and demonstrated in a cohort of 1,440 biopsies that GMI was predictive of graft loss, independently of histological diagnoses [28].

The presence of inflammatory cells in the peritubular capillaries (PTC) represents the second lesion in the category of MVI [3]. Hidalgo et al. performed IHC for CD3, CD68 and CD56 on 18 biopsies that were diagnosed as C4d positive-AMR, C4d negative-AMR and TCMR and found an increased number of CD68^+^ macrophages (p = 0.03) and CD56^+^ NK cells (p = 0.006) in the PTC in cases of AMR, independently of C4d staining, as opposed to TCMR [17]. In a study conducted by our group using multiplex IF, we found in the PTC a higher proportion of T lymphocytes during AMR and TCMR (81.1% and 87.6% respectively), than macrophages (14% and 10.5%, respectively) and NK cells (4.8% and 2.0%, respectively). However, the density of NK cells and macrophages were significantly higher in AMR compared to TCMR (4.7 ± 1.2 vs. 1.5 ± 0.5/mm^2^, p = 0.01 for NK cells and 11.6 ± 2.5 vs. 5.0 ± 1.5/mm^2^, p = 0.02 for macrophages) [19]. These results were not aligned with those from an older study by Liptak et al. that used electron microscopy and showed that monocytes represented 59% of cells in the PTC, while granulocytes and lymphocytes represented 14% and 12% respectively in a series of 12 AMR biopsies [26].

Computer-assisted counting of immune cells (CD20 for B lymphocytes, CD138 for plasma cells, CD4 or CD8 for T lymphocytes, CD56 for NK cells, FoxP3 for T regulatory cells, CD68 for macrophages with pSTAT1 or cMAF, to distinguish M1 and M2 macrophages respectively) using IHC on serial sections was used to characterize inflammatory infiltrates in different types of rejection [20]. Aguado-Dominguez et al. showed that T cells and non-polarized CD68^+^ macrophages represented 40% and 36%, respectively, of the total inflammatory cells found in the interstitium during AMR [20]. When further investigating T-cell subtypes, 21% were CD4^+^, 15% CD8^+^ and 4% FOXP3+. Even though this study analyzed only the interstitial compartment, clustering analysis revealed a correlation between NK cells and active AMR. Interestingly, they found that the cellular composition greatly varied across patients within the same diagnosis category, and failed to identify a unique profile associated with a given type of rejection [20].

Sicard et al. automatically quantified CD20^+^ cells, CD3 + cells, CD68^+^ cells and granulocytes using conventional IHC on serial slides in 52 AMR biopsies and showed that the extent of CD68^+^ macrophage infiltration was the sole predictive factor associated with subsequent graft function. The more intense the macrophage infiltrate in the interstitium and in the PTC the greater the rate of graft loss [21]. Furthermore, patients with a high macrophage density also had higher expression of C4d and a higher score of interstitial inflammation and tubulitis according to the Banff classification [21].

The prognosis value of macrophages probably results from their instrumental role in the priming and polarization of the adaptive immune response [29–31]. From a functional point of view, macrophages have been classically divided into M1 with a pro-inflammatory phenotype, while M2 macrophages are rather considered as anti-inflammatory and pro-fibrotic [32]. Using IHC in a cohort of 55 AMR samples, Kim et al. stained M1 and M2 macrophages using MRP8/14 and CD163 markers respectively [33]. They found that glomerular M2 macrophages were associated with chronic transplant glomerulopathy and poorer graft function, whereas tubulointerstitial M2 macrophages were associated with lower MVI and lower arteritis than the M1 polarization group [33]. The group also found a trend toward longer graft survival in patients that had higher numbers of glomerular M1 (p = 0.175) [33].

NK cell contribution to rejection has long been overlooked given the scarcity of lineage-specific markers to accurately differentiate NK cells from activated T cells [34]. Hidalgo et al. were the first to highlight the importance of NK cells during AMR by using data obtained by transcriptomics and CD56 IHC staining [17, 35]. However, it is worth to note that CD56 may be expressed by some T cell subsets as well, and the lack of CD3 expression by CD56-expressing cells should be requested to assign the label of NK cells with certainty. Although IHC was performed on a small number of patients with AMR (C4d positive and negative) and TCMR, the group highlighted a large increase of CD56^+^ cells in the peritubular capillaries of AMR patients when compared to TCMR (p = 0.03) [17]. Furthermore, they found a large number of NK-associated transcripts in biopsies that were done 1 year after transplantation with a diagnosis of either AMR of mixed rejection [35]. Moreover, in these biopsies, they found an important correlation between the presence of MVI, DSA positive status and NK specific transcripts [35]. In another study that used transcriptomic data and deconvolution analysis, obtained from 95 cases, 15 of whom had a diagnosis of AMR and 63 did not have rejection, Yazdani et al. found an increased number of NK cells in AMR cases compared to those without rejection [36]. Moreover, the presence of NK cells was correlated with MVI, DSA and C4d positivity. Out of all the cells types, NK cells were the best predictors of graft failure at 1 and 2 years, outperforming even the prognosis value of Banff classification (p < 0.001 vs. p = 0.039) [36]. Jung et al. used multiplex IF on a cohort of 39 for-cause biopsies (8 with no rejection, 11 TCMR and 20 AMR) and noticed that the highest density of NK cells was found in cases diagnosed with AMR (2.57 ± 2.58 cells/mm^2^) compared to 0.12 ± 0.28 cells/mm^2^ for non-rejection biopsies and 0.25 ± 0.34 cells/mm^2^ for TCMR (p = 0.002) [37]. Interestingly, the density of NK cell infiltrate was correlated with the “i” and “ti” scores as well as with the “ptc” (r = 0.489, p = 0.002), yet not with glomerulitis scores. In the study from Aguado-Dominguez et al., NK cells were mainly found in the cases of active AMR, whereas they were only a minor component in other types of rejection [20]. In a multiplex IF study conducted by our group on a cohort of 20 TCMR, 20 AMR and 5 non-rejection biopsies, we used the NK lineage-specific marker NKp46 to emphasize that NK cells represented only 2.7% ± 0.7% of the total inflammatory burden during AMR, as opposed to 0.6% ± 0.4% in normal biopsies and 2.9% ± 0.6% in TCMR [19]. More recently, Lamarthée et al. used single cell RNA-sequencing to show an increased density of FcγRIII+ NK cells in AMR and mixed rejection biopsies when compared to TCMR [12]. The same team also used deconvolution analysis of bulk transcriptomics data to demonstrate that NK cells and CD14^+^ monocytes/macrophages are more common in DSA+ AMR cases, whereas CD4^+^ memory T cells are more represented in DSA- AMR cases [11].

Graft-infiltrating B cells seem to play a minor role in the setting of active AMR, although a few studies have suggested an accumulation of B cells in the tertiary lymphoid structures that can develop in chronically rejected allografts [38]. This finding will be addressed later on. Aguado-Dominguez et al. showed that B cells represented 10% of the total interstitial inflammatory infiltrate [20]. In a multiplex IF study performed by our group from 125 rejection kidney biopsies, including 69 AMR, B cells accounted only for 3.4% of the infiltrating inflammatory cells (M1 and M2 macrophages, NK cells, T and B lymphocytes) during AMR (*unpublished data*). Importantly, the presence of CD20^+^ B cells did not correlate with positive C4d staining, suggesting that the presence of CD20^+^ cells in the allograft was independent of the presence of circulating DSA, produced by bone marrow or spleen-resident plasma cells [39, 40]. In another study, based on mRNA gene expression profiles in 21 cases with early AMR (diagnosed on average on the 9th day post-surgery), Viklicky et al. found that biopsies with a low expression of CD20, FoxP3, and TGF-β1 had an increased risk of graft failure in the next year [41].

## Chronic Active AMR

Chronic active AMR (CA AMR) is suspected when there is persistent, ongoing MVI with added features of transplant glomerulopathy (TG) and lamellation of the lamina densa of PTC as demonstrated by electron microscopy [3]. The exact mechanisms that lead to this pattern of injury are not yet fully understood [42], although recent studies that looked at gene expression profiles are starting to decipher the involved molecular pathways [43]. Adam et al. studied a panel of 34 genes in 197 non-human primates renal transplant biopsies and found 3 endothelial genes (VWF, DARC, CAV1) that correlated with the development of chronic glomerulopathy [44]. Interestingly, expression of these 3 genes was associated with C4d positivity (p < 0.001) and DSA positivity (p < 0.001) when compared to C4d negative and DSA negative cases [44]. Another study, based on gene expression profiling of chronic AMR, identified genes suggestive of NK cells, cytotoxic lymphocytes and activation of macrophages [45]. Interestingly, in this study, C4d-negative DSA-negative TG biopsies exhibited higher expression of cytotoxic T cell-associated transcripts, in keeping with enhanced T cell activation. A very recent study, based on the use of bulk RNA-sequencing, reported a significant increase in NK cell cytotoxic and T cells transcripts in biopsies with chronic AMR when compared to active AMR. Moreover, this study showed that CA AMR shared molecular features with TCMR, whereas neutrophils and monocytes-related pathways were predominantly involved in active AMR [46]. Deconvoluted RNA-sequencing data analysis also unveiled that the proportion of NK cells *in situ* was higher in CA AMR than in active AMR (p = 0.0038).

Recently, Cristoferi et al. investigated the differences between graft biopsies with either the diagnosis of TG C4d-/DSA- or TG C4d+/DSA+, through multiplex IF and bulk transcriptomics [47]. In line with the conclusion drawn by an above-cited study [46], C4d-/DSA-cases had higher numbers of CD3^+^ T cells and a higher expression of cytotoxic T-cell-associated mRNA than their C4d+/DSA+ counterparts. In contrast, the C4d+/DSA+ group had a predominance of infiltrating macrophages, NK cells and neutrophils [46, 48]. In the above-cited study from Aguado-Dominguez et al., 18 biopsies were diagnosed with CA AMR and disclosed an increased number of T cells and macrophages in the interstitial and glomerular compartments, with 39% of CD4^+^ T lymphocytes, 18% of CD8^+^ 18%, 6% of M2 macrophages, 4% of M1 macrophages and 2% of FOXP3+ cells [20]. CD138+ plasmocytes were also readily detected in CA AMR, unlike in active AMR (p < 0.05).

Papadimitrou et al. studied the cellular composition of glomerulitis in 240 transplant biopsies performed after 1 year post transplantation using IHC for CD3, CD20 and CD68 and its impact on TG’s outcome. They found a predominance of CD68^+^ macrophages, followed by CD3^+^ T lymphocytes. CD20^+^ B lymphocytes were barely identified. A high number of CD68^+^ macrophages (more than 12 in the most inflamed glomerulus) was strongly associated with TG, DSA and C4d [49]. Furthermore, the degree of macrophage infiltration in the glomeruli was also a strong predictor of subsequent graft dysfunction prompting the authors to hypothesize that the development of transplant glomerulopathy is preceded by the accumulation of macrophages. However, other studies have shown that T cells can also lead to transplant glomerulopathy in the absence of circulating DSA [47, 50].

Sablik et al. studied 20 biopsies with CA AMR using multiplex IF and evaluated T-cell subsets (CD3, CD8, FoxP3, Granzyme B), macrophages (CD68 and CD163), B cells (CD20) and NK cells (CD57) in the glomeruli (cells/glomeruli) and the tubulointerstitial compartment [cells/high-power field (HPF)] [51]. In the glomeruli, the main cell types were CD3^+^ T cells and macrophages, with an average of 5.5 cells and 4 cells per glomerulus, respectively. CD8^+^ T cells represented 61.7% of the total T cell population. Approximately 46% of CD8^+^ T cells and 23% of CD4^+^ T cells also expressed granzyme B, showing cytotoxic potential of these cell populations. NK cells, Treg and B lymphocytes were rarely found in the glomeruli. In the tubulo-interstitial compartment, the majority of cells were CD3^+^ cells with a mean number of 116.3 cells/HPF, followed by macrophages (21.5 cells/HPF). Interestingly, B cells aggregates were frequent in the tubulo-interstitial compartment. Unexpectedly, patients with a lower density of Treg in this compartment had a longer graft survival than patients with a high density (5.3 years vs. 2.1 years, p = 0.004).

## Acute and Chronic Active TCMR

In cases of acute TCMR, T cells are known to predominate and to drive the inflammatory response [13]. However, as for AMR, there are a number of other different immune cell types (macrophages, B cells, plasmacytes, NK cells, dendritic cells) that can be found, with different effects on the severity and outcome [19]. Moreover, Girlanda et al. demonstrated that T cell accumulation did not correlate with the extent of graft dysfunction, whereas monocytes did, suggesting that other immune effectors could be involved than cytotoxic T cells [13]. Hancock et al. reported a large number of macrophages in the tubulo-interstitial compartment, accounting for 52, 38, and 60% of the total infiltrating cells in mild, moderate and severe episodes of TCMR, respectively [52]. Similarly, Bergler et al. showed that CD68^+^ macrophages-rich infiltrates were found in severe cases of TCMR, associated with arteritis [14]. Furthermore, in this latter study, increased densities of macrophages correlated with reduced graft function at 3-year post transplantation [14]. Multiple studies have also shown that CD68^+^ macrophages infiltration positively correlates with the extent of interstitial fibrosis/tubular atrophy and with subsequent graft function [16, 53, 54].

**FIGURE 1:**
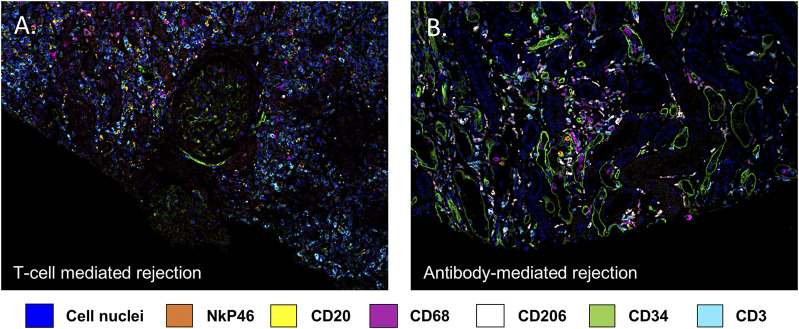
Multiplex immunofluorescence image highlighting the diversity of immune cells involved in kidney allograft rejection. Endothelial cells are stained with an anti CD34 antibody (green), B lymphocytes by an anti CD20 antibody (yellow), T lymphocytes using an anti CD3 antibody (turquoise), macrophages using an anti CD68 antibody (purple) and NK cells using an anti NkP46 antibody (orange). DAPI (blue) stains for cell nuclei. **(A)** illustrates a T cell mediated rejection, **(B)** illustrates an antibody mediated rejection.

**FIGURE 2:**
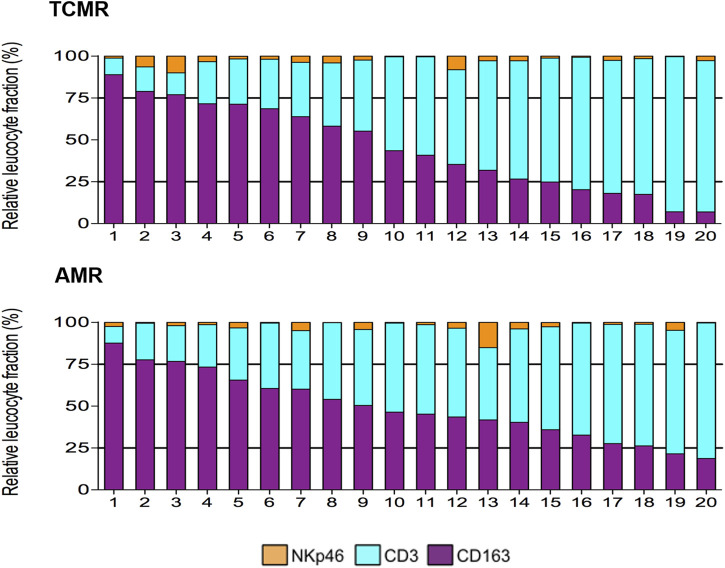
Heterogeneity of the composition of the inflammatory infiltrate during TCMR and AMR (from [19]). During both AMR and TCMR, biopsies displayed a wide range of proportions of the main inflammatory cells (CD3^+^ T lymphocytes, and CD163+ macrophages), and a small proportion of NkP46+ NK cells.

Using multiplex IF, our group has also shown that CD163+ macrophages are the second most common cell type (45.3% ± 5.8%) in TCMR after CD3^+^ lymphocytes (51.8% ± 6.0%) [19]. A representative image from a case from this study is depicted in Figure 1. Furthermore, we showed a great heterogeneity in the composition of the cellular infiltrate across the 20 individual patients with TCMR. As a matter of fact, the frequency of macrophages ranged from 7.0% to 89.0% while the frequency of CD3^+^ T lymphocytes varied from 10.0% to 92.7% of the total leukocytes infiltrating the graft [19]. On the other hand, we identified remarkable similarities, regarding the composition of the infiltrates, between patients with different pathological diagnoses (TCMR and AMR), as highlighted in Figure 2 [19]. We failed to identify any clinical or pathological factors that could predict the proportions of CD3^+^ T lymphocytes and macrophages in this series [19].

Macrophage accumulation in areas of interstitial fibrosis and tubular atrophy indicates a tissue repair-related universal phenomenon, independent of the pathogenesis process, with M2 outnumbering M1 [16, 32]. Notably, macrophages gain the ability to produce profibrotic mediators through M1 to M2 phenotype shift [30]. An IHC study by Ikezumi et al. showed that the number of infiltrating M2 CD68^+^ CD163+ macrophages increased over time after transplantation and correlated with the loss of glomerular filtration rate (p < 0.0001) as well as with the extent of interstitial fibrosis and tubular atrophy (p < 0.0001), whereas T cell accumulation did not [32].

Regarding B lymphocytes, using IHC Hwang et al. revealed CD20^+^ clusters (defined by more than 275 cells/HPF) in 37.3% of patients diagnosed with TCMR [55]. The presence of CD20^+^ clusters seemed to be associated with a poor graft survival and with steroid resistance [40, 56, 57]. On the other hand, other studies have yielded conflicting results with CD20^+^ infiltrates having no effect or even being associated with a better allograft survival [58, 59]. The implications of B-lineage cells in kidney allograft have been superbly reviewed by Filippone EJ and Farber JL [39].

Plasmocytes have also sparkled interest primarily in the context of plasma cell-rich TCMR, but also in some cases featuring AMR or mixed rejection lesions [60, 61]. Currently, this type of rejection is not individualized *per se* in the Banff classification, although an infiltrate with more than 5%–10% plasmocytes should be acknowledged by an asterisk after the inflammation score “i” [3]. Plasma cell-rich rejection is usually defined by the presence of plasmocytes in more than 10% of the cortical surface [60]. Using IHC, Mubarak et al. found plasma cells accumulating in the periglomerular area, in the perivascular space as well as at the cortico-medullary junction [62]. Interestingly, the number of plasma cells inversely correlated with the number of B cells [62]. The frequency of this rejection pattern varies between 2% and 14% [63]. Some studies have suggested that infections and poor adherence to the treatment can be risk factors for developing a plasma cell-rich infiltrate [64, 65]. Unfortunately, there is no standard treatment for this type of rejection and therefore these cases are usually refractory to treatment and have a very poor prognosis [60, 63]. These patients usually have low graft survival, with 40%–60% of cases promptly losing their graft following the diagnosis of plasma cell-rich episode of rejection [60, 64]. When considering the diagnosis of plasma cell-rich TCMR, the BK virus nephropathy is an important alternative diagnosis to rule out, since half of BKV-related renal inflammation exhibits plasma cell-rich infiltrates [66].

Over the past years, innovative tools have allowed a deeper understanding of the cellular composition during TCMR. Salem et al. were the first team using spatial transcriptomics in a single case of chronic active TCMR compared to a control case with no rejection [23]. The analysis focused on 5 regions of interest within the tubulo-interstitial space and emphasized an increase in genes related to T lymphocyte proliferation and activation. Interestingly, these findings were not correlated with the lesions scored according to the Banff classification. The authors also studied 3 glomerular areas and found no differences between controls and chronic active TCMR. In mice, single cell RNA-sequencing data obtained by Shen et al. showed that the inflammatory infiltrate evolves along with the progression of the rejection from the acute to the chronic phase. More specifically, the proportion of B cells, neutrophils and CD8^+^ T cells decrease over time, while macrophages become more prevalent [67]. Another single cell RNA-sequencing study performed by Liu et al. performed on 2 biopsies with chronic lesions showed an increase in memory B cells, myofibroblasts and activated monocytes [68].

Recently, Vaulet et al. studied the infiltration of 9 different immune cells by investigating 3 different data sets of 909 biopsies obtained by bulk transcriptomics. This study highlighted that the greatest amount of infiltrating inflammatory cells was observed in TCMR, whereas DSA+ AMR, in contrast, had the lowest number of infiltrating cells, close to that of non-rejection biopsies [11]. When compared to the non-rejection group, TCMR cases had an increase of 16.5% (p < 0.001) in the number of inflammatory cells. Furthermore, there was an increase in CD4 naive cells (+1.8%, p < 0.001) and CD14^+^ monocytes/macrophages (+5.9%, p < 0.001) when compared to non-rejection cases. NK cells had a lower contribution to the infiltrates when compared to AMR cases, while CD8 effector cells demonstrated the greatest frequency in TCMR and DSA-positive mixed rejection. Interestingly, the main Banff rejection diagnosis categories could not be individualized based on the estimation of immune cell composition alone.

Zhou et al. also showed interesting differences between cases diagnosed with TCMR and stable graft biopsies by analyzing transcriptomic data available from the public domain, obtained from 224 TCMR and 1,561 stable samples [69]. Similarly, as in the study from Vaulet et al. [11], they estimated the relative proportion of 22 immune cell types by deconvolution analysis. The investigators found that biopsies with TCMR had a reduced infiltration by naïve B cells, M0 macrophages, neutrophils and resting dendritic cells, yet an increased proportion of memory B cells, CD8^+^ T cells, CD4^+^ T cells, follicular helper T cells, gamma delta T cells, monocytes, M1 macrophages, activated dendritic cells and eosinophils when compared with biopsies free of rejection [69].

## Other Minor Components of the Inflammatory Infiltrate

### Tertiary Lymphoid Organs

B cells may aggregate and form tertiary lymphoid nodules in the allograft which are composed of B lymphocytes, follicular dendritic cells (FDC), T follicular helper (TFH) cells and a rim of T lymphocytes, plasmocytes and plasmablasts [39]. These structures are supported by lymphoid vessels and high endothelial venules [39]. The main goal of these structures is to form antibodies after an interaction between TFH cells and B cells [70]. They have been proven to be very important in different scenarios such as autoimmunity, cancer and infection [71]. However, the study of lymphoid nodules is complicated by a lack of standardization and by the fact that the classification of these structures according to the Banff criteria is difficult [3]. Therefore, to date, it is not clear whether accurate diagnosis or treatment is needed in order to disrupt the formation of tertiary lymphoid organs, as they seem to have some potential to induce tolerance of the graft [39].

In the setting of kidney transplantation, tertiary lymph node formation has been demonstrated in acute and chronic rejection scenarios [38]. Using IHC, De Leur et al. showed that ectopic lymphoid structures were predominantly found in acute TCMR, unlike in AMR [70]. In a series of 26 cases of explants, 20 of which were diagnosed with chronic rejection, Thaunat et al. found tertiary lymphoid structures in almost all cases of chronic rejection [72].

Lee et al. showed in a large series of 214 patients with protocol biopsies without evidence of rejection that almost half of the biopsies (46.9%) had aggregates of lymphoid cells classified as tertiary lymphoid structures that formed as early as within the first month after transplantation [73]. Interestingly, only 3.8% of implantation biopsies demonstrated such structures. The further development of stage II tertiary lymphoid tissues, defined by the presence of FDC in these structures was gradual, from 1.4% in 0-h biopsies, to 3.6% at 1 month and 18.9% at 1 year [73]. In this cohort, the finding of FDC correlated with a subsequent decay of the graft function as well as with the presence of DSA, even though no patient developed a subsequent episode of AMR. However, other studies did not confirm the association between FDC and rejection, but in the contrary, suggested that tertiary lymphoid nodules could play a role in graft tolerance [74]. Using a mouse model of kidney allograft tolerance, Brown et al. demonstrated using IHC the presence of tertiary lymphoid structures in these kidneys [75]. They further showed that there was a mild correlation between the size of the lymphoid structures and graft function, with larger sized nodules being seen in better functioning grafts [75].

### Mastocytes

Mastocytes represent a very versatile cell type, with the capacity to both increase or decrease the inflammatory processes that takes place during rejection, depending on whether they secrete anti-inflammatory factors or degranulate pro-inflammatory mediators. Their significant impact on the course of the inflammatory response contrasts with their minor contribution to the infiltrate [76]. Outside the field of transplantation, mastocytes have mostly been involved and described in the setting of allergy [77]. Regarding transplantation, they have been observed as a component of acute rejection, although not in all studies [76–78]. It is also hypothesized that mastocytes, recruited by Treg-produced IL-9 [79], could be involved in the maintenance of allograft tolerance, although the exact mechanisms remain ill-defined [76].

In a study performed by Varol et al. using IHC for tryptase in 53 biopsies diagnosed with borderline TCMR, mastocyte accumulation was correlated with delayed graft function (p = 0.020) and deceased donor status (p = 0.035) [77]. The authors found an average of 10.79 mast cells/mm^2^ in the interstitial space of the cortex, with almost no mastocytes being found in the glomerular or vascular compartments [77].

While there are not many studies, mastocytes have been proven in some series to be important in the setting of chronic rejection, with the levels of mastocytes correlating with the extent of interstitial fibrosis and tubular atrophy, with the decline in graft function and also with the time after transplantation [80–82]. In a transcriptomic study from the Edmonton group that analyzed 129 for-cause biopsies from 104 patients, it was shown that there is a correlation between the levels of mastocyte-associated transcripts and the extent of chronicity Banff scores as well as a worse graft prognosis [83]. Moreover, biopsies that had a low level of mast cell transcripts had a better graft survival.

### Eosinophils

Eosinophils are usually considered as aggressive cytotoxic leukocytes involved in the innate defense system, seen in diverse conditions such as allergic diseases, autoimmune diseases and parasite infection [84]. Their scarcity in kidney biopsies makes them difficult to study and therefore, their exact role in alloimmunity has remained controversial and poorly understood [85].

In the context of acute rejection, their role remains uncertain although some reports find a connection between blood eosinophilia and the diagnosis of rejection [84, 86]. Moreover, old studies have shown accumulation of eosinophils in the graft in a context of vascular rejection, although conflicting results have been published since then [87–89]. In an older study reported by Hongwei et al. using manual counting and a carbol chromotrope staining protocol, the density of eosinophils was much higher in cases with acute rejection (0.4–1.1 cells/μm^2^) when compared to cases with no rejection (less than 1 cell/μm^2^). Interestingly, biopsies diagnosed with rejection that progressed to graft loss had a higher density when compared to those who did not (1.9 vs. 0.2 cells μm^2^, p = 0.014) [88]. Vanikar et al. showed in a more recent study performed on 1,217 kidney transplant biopsies by using hematoxylin and eosin-stained slides that the presence of tissue eosinophilia (defined as ≥4% eosinophils in the interstitium) was associated with poor graft outcomes [90].

In a study done by Nolan et al. on allograft nephrectomy specimens using epifluorescence, the authors highlighted the presence of eosinophils in the intima, in the adventitia of vascular walls and in the interstitium in 73%, 80%, and 87% of chronic rejection cases respectively [91]. Furthermore, they showed that the medium from cultured eosinophils stimulates DNA synthesis of vascular smooth muscle cells, therefore indicating a potential role of eosinophils in the development of chronic vascular lesions in the allograft [91].

### Neutrophils

Neutrophils can be seen as a link between the innate and the adaptative immunity [92]. Furthermore, they can elicit opposite functions in the immune response, from one extreme (anti-inflammatory and regulatory) to the other (pro-inflammatory) [92]. In acute rejection, neutrophils are activated by endothelial cells and then, after crossing the vessel walls, are involved in the destructive release of reactive oxygen species or in programmed cell death [93–95]. Neutrophil depletion experiments have indeed revealed the importance of neutrophils in promoting alloimmune responses. For example, in a mouse skin transplant model neutrophil depletion mitigated the acute rejection by attenuating the recruitment of alloreactive memory CD8^+^ T cells [96]. Neutrophils may stimulate the recruitment of activated CD8^+^ T cells through their expression Fas ligand, which can induce expression of the T cell chemoattractant CCL1, CCL2 and CCL5 [97]. In AMR, little is known about the mechanisms of neutrophil activation, even though neutrophilia has been observed as a sign of ongoing rejection [92]. The Banff classification specifies that an asterisk shall be added to Banff Lesion Score “i” (e.g., “i1*”), if there are more than 5%–10% of eosinophils, neutrophils or plasma cells [4].

In the setting of chronic rejection, neutrophils can sometimes be observed, but unfortunately data regarding their proportion and the exact mechanisms by which they promote inflammation are poorly described [92]. The current view is that neutrophils accumulation is driven by IL-8 and IL-17-dependent chemotactic pathways, and get activated by the exposure to damage associated molecular patterns [98, 99].

### Follicular Dendritic Cells

FDCs are the most effective antigen presenting cells in mice and humans and can be found in both lymphoid and non-lymphoid tissues [100]. In the kidney, as for other tissues, FDCs are derived from bone marrow-derived hematopoietic stem cells [101]. Until now, only a few studies investigated the link between the presence of FDCs in transplanted kidneys and the allograft survival. Using IHC, Batal et al. stained CD209+ DCs in 105 allograft biopsies from kidney transplant recipients with various diagnosis (TCMR, AMR, mixed rejection and others) [102]. They found an association between a high dendritic cell density and a poor graft survival and localized these cells mainly in the interstitium, occasionally in the peritubular capillaries and rarely in the tubules, glomeruli or arteries [102]. Yazdani et al. found an increase in FDCs-associated genes in AMR and TCMR compared to patients without rejection, but no differences between AMR and TCMR patients [36]. In a murine model of kidney transplantation, Zhuang et al. confirmed that donor DCs were mainly replaced by recipient FDCs originating from non-classical monocytes 7 days after transplantation [103]. Depletion of these recipient FDCs by diphtheria toxin significantly prolonged graft survival compared to controls injected with PBS [103]. Although studies on animal models shed some light on the function of FDCs, studies on human kidney allografts are still lacking and will be necessary to truly understand their role in kidney allograft rejection.

Another aspect of FDCs is their capacity to induce rejection but also tolerance. Tolerogenic FDCs, also called FDCregs, can suppress the function of T cells or provide a weak stimulation. They are also involved in the generation of induced Tregs [104]. Therefore, cell-based immunotherapy with tolerogenic FDCs is now recognized as a promising approach to increase the survival time of grafts and to reduce the use of immunosuppressor treatments. Moreau et al. published in 2023 the results of the first phase I/II clinical trial using autologous tolerogenic FDCs (ATDC) immunotherapy in kidney transplant recipients [105]. Eight patients received ATDC the day before transplantation in conjunction with standard steroids, mycophenolate mofetil and tacrolimus immunosuppression. The control group composed of 9 patients received the same standard immunosuppression, with ATDC replaced by basiliximab induction. In both groups of patients no death occurred, the graft survival was 100% at 3 years and there were no adverse events related to ATDC infusion. Furthermore, monitoring of circulating immune cells in patients reported no increase of activated CD8 T cells in the ATDC group when compared to the reference group [105].

Involvement of FDCs in ischemia-reperfusion, rejection and tolerance makes them difficult to characterize and their role in each process still needs to be clarified. Upcoming studies will have to carefully choose the markers used to discriminate all the subpopulations of FDCs to clearly identify their specific role in each process. In rejection, a better characterization of their localization inside the nephron will also help to disclose their contribution to the alloimmune response.

## Impact on Treatment

The incidence of diagnosed TCMR episodes has significantly decreased due to advances in effective therapeutic options. Consequently, research efforts have shifted toward addressing AMR [5]. Despite ongoing advancements in our understanding of AMR, the development of novel therapies targeting acute episodes and preventing chronic lesion formation remains limited [106]. As highlighted earlier in this review, AMR involves a variety of cellular types, contributing to its complexity. This cellular heterogeneity presents both opportunities and challenges for the development of effective therapies.

Notable progress has been made in targeting AMR, particularly with felzartamab, an anti-CD38 monoclonal antibody that acts on plasma cell and NK cells [107]. In a recent trial, 22 patients diagnosed with AMR were randomized to receive either felzartamab or a placebo. After 24 weeks, patients in the felzartamab group demonstrated significantly lower MVI scores (0 vs. 2.5) and reduced levels of donor-derived cell-free DNA (0.31% vs. 0.82%), compared to the placebo group [107]. Although these promising results have sparkled significant interest in CD38 targeting strategies, they have also raised critical questions regarding the underlying mechanisms of action. Notably, the marked improvement in intra-graft inflammation scores observed with treatment contrasts sharply with the minimal, if any, effect on DSA levels. This discrepancy suggests that the therapeutic efficacy may not primarily rely on modulating antibody responses, but rather on targeting other CD38-expressing effector cells, with NK cells emerging as main culprits. Further research is needed to elucidate the composition of AMR-associated inflammatory infiltrates before and after anti-CD38 therapies.

The interleukin-6 (IL-6) signaling pathway, which is critical in the maturation of B cells into plasma cells, has also been explored as a therapeutic target in AMR [108]. For instance, clazakimumab, an anti-IL6 antibody, was tested in patients with CA AMR. However, the trial was discontinued due to lack of efficacy [108, 109]. Additionally, a humanized anti-IL-6 receptor antibody, tocilizumab, is currently being evaluated in the INTERCEPT trial, which involves 50 patients with an established diagnosis of CA AMR [110].

## Should We Still Classify Rejection Into Cellular or Humoral?

**FIGURE 3:**
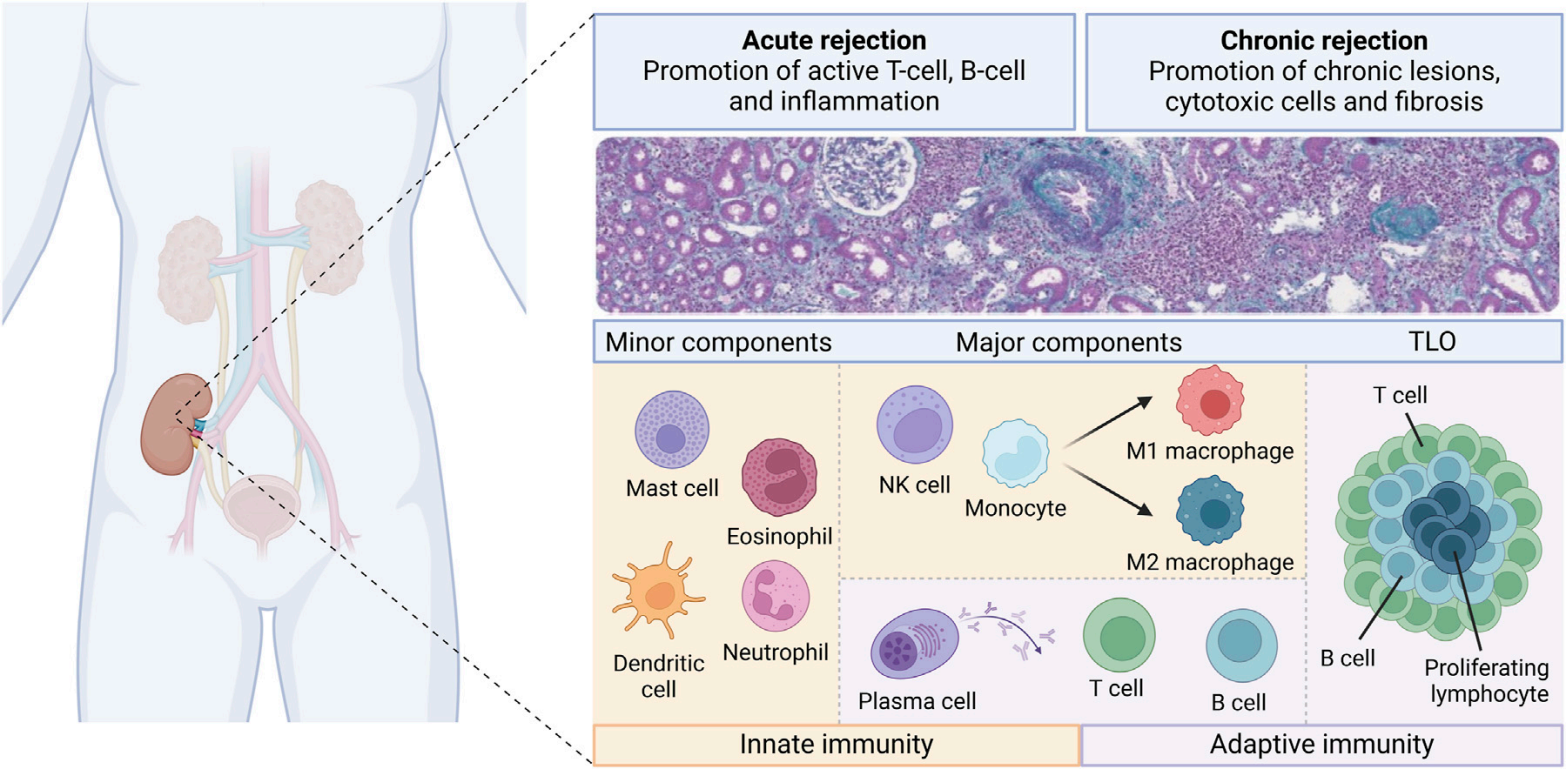
Overview of the diversity of the immune cell landscape during kidney allograft rejection. Different immune cells from innate (orange boxes) and adaptive (purple boxes) immunity are involved during the course of kidney transplant rejection. There are classified as minor components, major components and tertiary lymphoid organs (TLO). These components may play a role into acute and chronic rejection, whether in T-cell mediated rejection or antibody-mediated rejection.

The current literature demonstrates that many immune cell types with various densities and proportions are involved in kidney allograft rejection (Figure 3). Different patterns of injury can be histologically distinguished, according to the localization of the cells (i.e., within the capillaries or mainly within the interstitium). However, several studies have demonstrated that the cell type composition and the molecular pattern may be similar across different histological types of rejection. Furthermore, for the same type of histological rejection, the nature and the proportion of the cells may be highly variable from an individual to another [19]. Given the fact that the presence or absence of different cell types may carry significative prognostic impact, this raises the question whether we should classify rejection only based on the localization of the cells (i.e., in the interstitial compartment or in the vascular compartment) rather than on the type and quantity of cells involved. For example, Azad et al. described a panel consisting of 3 genes that were common for both AMR and TCMR and that correlated with the degree of injury [111]. Using transcriptomic data obtained from 1,571 renal biopsies, the group found that pro-inflammatory macrophages correlated with the presence of a common rejection module [111]. This suggests that there are common pathways in both TCMR and AMR, further questioning the rationale and somehow arbitrary separation between these 2 types of rejection. In the transcriptomic study from Shah et al., the authors showed overlap and differences in the genes expressed in different rejection phenotypes [46]. Active AMR, chronic AMR and TCMR shared 117 genes while expressing also 231, 60, and 114 different genes respectively when compared to normal biopsies [46]. Interestingly, this study has shown that there are more similarities between chronic AMR and TCMR than between chronic and active AMR [46]. Furthermore, as already mentioned, our previous study using multiplex IF has shown similar composition of CD3^+^ T cells, NK cells and CD163+ macrophages in biopsies with different types of rejection [19].

In conclusion, as different types of transcriptomic data and cell counting techniques emerge and become more readily available, further studies will probably further elucidate the common and different pathways encountered in different rejection settings, and help to better understand the pathophysiology of rejection. Moreover, the precise description of molecular pathways and cells involved in rejection episodes may help assess the prognosis more accurately after rejection and guide treatment, by developing cell-specific therapies rather than global immunosuppressive therapies. Whether cellular composition will be implemented in the Banff classification to refine the categorization of different rejection types has yet to be clarified.
